# Tax on Professions

**Published:** 1877-05

**Authors:** 


### TAX ON PROFESSIONS.

The laws in Georgia were thought to be oppressive and unjust when the whole revenue of the State was obtained by special tax, without strict regard to the value of property. Land, for instance, whether rich or poor, was taxed a mere nominal amount per acre, while the bulk of taxes was raised from other sources.

Finally, the *ad valorem* system was adopted, but very imperfectly, as will be seen by reference to the large number of items for special tax. Physicians, lawyers, etc., are made to pay a certain specific amount, in addition to the advalorem on property and income. This, it may be urged, is just and proper, on account of the protection afforded by special license from the State; but it must be remembered that the license serves, and was intended, for public protection, and not for the person licensed. Why is it that a lawyer or doctor must stand an examination before a board legally appointed, before he can be entrusted with the life or property of individuals? Certainly, not to protect himself against the rivalry of others who may seek to become his competitors in business, but to insure his competency to discharge the duties of his profession to patient or client. If, then, any debt of gratitude or money is due the State for the protecting influence of license, it is certainly from the public, or those served by the professions, rather than from the professions themselves.

The resolution on this subject, passed by the Georgia Medical Association at its recent meeting in Macon is, we think, a move in the right direction. The physician who serves the public faithfully, and, in some instances, gratuitously to the amount of one-fourth of his year's labor, should not be subjected to the onorous State and municipal taxation that is imposed on him. We claim nothing for the work of charity bestowed on those unable to pay, but we do think that this poor privilege of working for the public for nothing and supporting ourselves should entitle us to equality, at least, with other people in point of taxation. Practicing physicians of this city were once subjected to ten or twenty dollars tax by the United States government, ten by the State, and at one time twenty-five by the city; all of which amount to, at least, forty-five dollars per annum. All this was assessed in addition to the regular tax on property and income, which physicians pay like other people.

Now, this is simply monstrous, unjust, and oppressive. Moreover, while this unjust discrimination is made against physicians in the matter of taxation, they are denied the advantages of merchants and others in the collection of debts from those who seek to defraud them. While certain other classes of creditors are allowed to garnishee employers to secure a debt, physicians are debarred this privilege.

We hope the profession will assert their rights, in a manner to be heard and recognized in the halls of legislation.

